# The Role of Nutrition in Exercise and Sports

**DOI:** 10.3390/nu18091354

**Published:** 2026-04-24

**Authors:** Roberta Ceci, Guglielmo Duranti

**Affiliations:** Laboratory of Biochemistry and Molecular Biology, Department of Movement, Human and Health Sciences, Università degli Studi di Roma “Foro Italico”, Piazza Lauro de Bosis 6, 00135 Rome, Italy; roberta.ceci@uniroma4.it

Nutrition affects all bodily processes by supporting energy production and tissue maintenance. Proper adaptation to exercise depends on balanced macro- and micronutrient intake to optimize performance, recovery, and overall health. Nutritional needs vary by sport, individual characteristics, and environment. Dietary supplements are often used to enhance performance, recovery, and adaptation, while reducing negative effects such as muscle damage, oxidative stress, and fatigue. As a result, sports nutrition is a constantly evolving field aimed at optimizing athletic performance through tailored strategies.

Interest in targeted nutrition for muscle health has grown significantly in recent years, reflecting the key role of skeletal muscle in overall well-being [1,2]. Skeletal muscle makes up about 40% of body weight and is essential for movement, posture, and metabolism. Because it is regularly exposed to mechanical and oxidative stress, it requires specific nutritional support depending on exercise type, intensity, and duration. This has led to an increase in research on exercise-related nutrition [3,4,5,6] (Figure 1).

Athletes’ nutritional needs vary according to training and competition phases and are influenced by energy demands, nutrient composition, and individual preferences. Hydration, protein intake, and supplement use may also differ. Proper nutrition not only meets energy requirements, but also provides micronutrients that protect muscles from stress. While moderate exercise promotes beneficial adaptations, intense activity can cause muscle damage, inflammation, and increased production of reactive oxygen species (ROSs), which impair cellular function and contribute to fatigue [7,8,9,10,11].

In the past two decades, research has increasingly focused on how nutrition can modulate ROS production and support muscle function, highlighting its central role in exercise physiology [12].

In this context, this Special Issue, titled “The Role of Nutrition in Exercise and Sports”, published in the *Nutrients* journal, was developed with the aim of collecting new research in the field, and has ultimately collected nineteen contributions—twelve original research articles, a perspective paper, a brief report, a meta-analysis and four reviews—offering new insights into the impact of nutrition on exercise-induced oxidative stress, muscle function and adaptation, with particular attention to physical activity.

Physical exercise can trigger a range of adaptations that help individuals cope with exercise-induced stress, and nutritional strategies can further support this process. Research indicates that prolonged or high-intensity training may temporarily impair immune function [13]; for instance, a marked reduction in the leukocyte-to-monocyte ratio has been observed. Some studies also report that intense training can diminish monocyte activity. Conversely, other findings suggest that physical activity may increase monocyte counts, potentially as part of muscle tissue remodeling in response to training-induced damage [14].

Zhang and colleagues demonstrated that sustained, high-intensity exercise can lower the leukocyte-to-monocyte ratio in young athletes, an effect that may be mitigated by spirulina supplementation. Spirulina is a dried biomass derived from *Arthrospira platensis*, often mistakenly referred to as a blue-green alga, although it is actually a cyanobacterium belonging to the order Oscillatoriales. It is a rich source of gamma-linolenic acid (GLA) and omega-3 and omega-6 fatty acids, and also contains alpha-linolenic acid (ALA), linoleic acid (LA), stearidonic acid (SDA), eicosapentaenoic acid (EPA), docosahexaenoic acid (DHA), and arachidonic acid (AA). In addition, spirulina provides several vitamins, including B1 (thiamine), B2 (riboflavin), B3 (nicotinamide), B6 (pyridoxine), and B9 (folic acid), as well as vitamins C, D, A, and E. Consequently, spirulina supplementation may help enhance immune defenses against parasites, pathogenic bacteria, and acute allergic responses (contribution 1).

In recent years, there has been growing interest in nitrate-rich foods, particularly in the field of sports performance. Among these, beetroot (from the *Chenopodiaceae* family) represents an important dietary source of nitrates [15]. In humans, beetroot supplementation has been shown to enhance performance during submaximal endurance exercise. For example, a higher nitrate intake (11.2 mmol) was found to reduce muscle fractional oxygen extraction by 19% and increase time to exhaustion during high-intensity cycling by 15.7% compared to a placebo [16].

Since improvements in exercise performance associated with beetroot-derived nitrate supplementation may result from enhanced delivery of oxygen and metabolic substrates to active skeletal muscle, it is important to extend these findings beyond endurance activities. Notably, because nitrate conversion to nitric oxide (NO) is facilitated under hypoxic and highly acidic conditions, beetroot supplementation could have even greater effects during sprint exercise, potentially accelerating recovery between efforts by improving oxygen availability [17].

Despite these promising findings, a meta-analysis by Wong et al. did not report significant improvements in peak or mean power output during repeated sprint protocols following either acute or chronic beetroot supplementation. However, substantial variability in study designs (including differences in exercise protocols, nitrate dosage, type of beetroot product, supplementation strategies, and intervention duration) may limit the ability to draw firm conclusions regarding its effects on power output in repeated sprint exercise (contribution 2).

Novel nutritional compounds have recently gained attention. One such compound, arginine silicate stabilized with inositol, has been shown to increase circulating levels of arginine and nitric oxide [18]. Nitric oxide, in turn, promotes vasodilation in peripheral tissues, as well as in the brain [19,20]. Maintaining adequate cerebral blood flow, along with sufficient oxygen and nutrient delivery, is considered essential for preserving cognitive function and brain health over time [19,20].

In this context, elevated nitric oxide levels have been associated with improvements in cognitive performance and learning through multiple mechanisms. These include enhanced potassium channel excitability, calcium-dependent activation of neuronal nitric oxide synthase, modulation of *N*-methyl-D-aspartate (NMDA) receptor activity via nitrosylation, and regulation of pathways involved in synaptic transmission [19,20]. Such effects may also be particularly relevant in sports settings.

Supporting this idea, Sowinski and colleagues demonstrated that acute supplementation with arginine silicate stabilized with inositol (ASI) combined with inositol (I) improved reaction time and working memory across several cognitive tests. Specifically, ASI + I supplementation enhanced both reaction speed and accuracy in a group of healthy, experienced gamers aged 18–40 years. Improvements were also observed in performance on the Sternberg test, including faster response times across tasks of increasing difficulty, compared to the placebo group (contribution 3).

The importance of carbohydrates in sports performance is well established. In sports nutrition, the use of mixed carbohydrate solutions (designed to take advantage of the distinct absorption properties of different carbohydrates such as glucose, maltose, oligosaccharides, and trehalose) has been proposed as an innovative strategy compared with traditional carbohydrate supplementation [21]. Among naturally occurring carbohydrates, trehalose (α-D-glucopyranosyl-α-D-glucopyranoside) is a disaccharide composed of two glucose molecules linked by an α,α-1,1 glycosidic bond [22]. It is found in foods such as mushrooms, yeast, and algae [23]. Although naturally present only in small amounts and therefore considered a rare sugar, the development of enzymatic production methods has enabled its large-scale, cost-effective manufacturing, facilitating its application in sports nutrition.

More recently, Hamada and colleagues investigated the effects of trehalose supplementation in 10 healthy male college students (aged 20–22) who engaged in at least 180 min of exercise per week. Participants consumed trial beverages containing 8%, 6%, or 4% trehalose after the first set of a Wingate test. The results showed no significant differences in performance during the final stages of a 5 h high-intensity intermittent exercise protocol among the three groups. Since performance outcomes were comparable across all concentrations, the lowest dose tested (20 g of 4% trehalose) was sufficient to sustain high performance during prolonged exercise.

Based on these findings, and considering the slower absorption profile of trehalose, the authors proposed a novel approach to carbohydrate supplementation: the formulation of mixed carbohydrate solutions that leverage differences in absorption rates, insulin responses, and sensory characteristics of various carbohydrates, such as glucose, fructose, and highly branched cyclic dextrin (contribution 4).

Carbohydrate ingestion is well known to enhance aerobic endurance by increasing the availability of exogenous substrates for oxidative phosphorylation, thereby helping to preserve muscle glycogen during prolonged, high-intensity exercise. More recently, an alternative strategy, carbohydrate oral rinsing, has emerged as a potentially effective approach for improving performance across different exercise modalities, including short-duration endurance activities (<1 h), resistance exercise, repeated sprints, and neuromuscular performance [24,25,26].

Karayigit and colleagues investigated the effects of carbohydrate mouth rinsing (CMR) in a group of sixteen healthy, resistance-trained males. Their findings showed that administering a 6% carbohydrate solution as an oral rinse three times improved performance in repetitions to failure at 80% of one-repetition maximum (1 RM), likely through centrally mediated mechanisms. However, no significant performance enhancement was observed under a lower-intensity condition (40% of 1 RM).

Based on these results, the authors suggest that the effectiveness of carbohydrate oral rinsing depends on factors such as exercise intensity and duration. Consequently, coaches and athletes should apply this strategy selectively, using it to optimize performance, particularly in high-intensity muscular endurance contexts (contribution 5).

Skeletal muscle relies heavily on glycogen stores as a rapid source of carbohydrates. Indeed, muscle glycogen represents a critical energy substrate during high-intensity exercise and plays a central role in the development of fatigue [27]. Consequently, the identification of effective nutritional strategies to promote rapid recovery and glycogen resynthesis following intense exercise is a key objective in sports nutrition.

In this context, recent evidence suggests that several bioactive compounds of natural origin may support glycogen metabolism.

It was demonstrated that polyphenol supplementation may support cellular metabolism and mitochondrial biogenesis in athletes, potentially delaying fatigue onset and enhancing performance [28,29]. However, the findings are not always consistent. For example, Huang and colleagues reported that four days of oral resveratrol supplementation in nine healthy young male participants (mean age 20.2 ± 0.4 years; VO_2_max 51.4 ± 1.7 mL/kg/min) did not significantly affect fat oxidation during 60 min of cycling at 70% VO_2_max, and nor did it enhance muscle glycogen resynthesis.

Similarly, no improvements were observed in insulin sensitivity or in the expression of key genes involved in skeletal muscle glucose metabolism, including TBC1D1, TBC1D4, GLUT4, and HKII. In addition, resveratrol supplementation did not upregulate genes associated with mitochondrial biogenesis in skeletal muscle during recovery, such as SIRT1, PGC-1α, ERR-α, NRF1, NRF2, and TFAM (contribution 6). The authors suggest that these findings may be explained, at least in part, by the dosage employed, as has been observed with other nutritional supplements.

Polyphenols, along with other bioactive compounds found in foods, may contribute to mitigating exercise-induced oxidative stress. A growing body of evidence suggests that moderate levels of reactive oxygen species (ROSs) and inflammation generated during exercise can promote beneficial muscular adaptations, including enhanced endogenous antioxidant defenses, mitochondrial biogenesis, and tissue regeneration [30]. In contrast, excessive ROS production can exert harmful effects at the cellular level.

As a result, antioxidant and anti-inflammatory nutrients have attracted considerable interest as potential strategies to counteract exercise-induced oxidative stress and inflammation [31]. Among these, vitamin E plays a key role as a lipid-soluble antioxidant and chain-breaking agent, interrupting lipid peroxidation and preserving the integrity of polyunsaturated fatty acids within cell membranes [32].

In a meta-analysis presented in this Special Issue, Kim and colleagues reported that dietary vitamin E supplementation significantly reduced biomarkers associated with exercise-induced muscle damage and oxidative stress. Specifically, its protective effects were evident in reductions in creatine kinase (CK) and malondialdehyde (MDA), which were elevated immediately after exercise in non-supplemented athletes. Notably, even low-dose vitamin E supplementation (≤500 IU/day) demonstrated significant protective effects.

However, as commonly observed with antioxidant supplementation, these effects appear to be highly dependent on dosage, timing, and the magnitude of exercise-induced stress. Importantly, the authors also noted that vitamin E supplementation did not produce significant effects on exercise-induced inflammation (contribution 7).

In certain sports, nutritional requirements are especially demanding. Combat sports, for instance, involve repetitive and high-intensity actions such as striking (kicks, punches, and defensive blocks), grappling (takedowns and submission techniques), and combinations of both, all of which require substantial energy expenditure. Moreover, these disciplines carry an inherent risk of injury, with most athletes experiencing at least one injury over the course of their careers. Since injuries are difficult to avoid entirely despite preventive measures, optimizing treatment and rehabilitation strategies to reduce recovery time represents an important area of research.

Beyond its impact on physical and psychological well-being, nutrition plays a central role in athletic performance, injury risk, and recovery outcomes [33,34]. In this regard, Turnagöl and colleagues provided a comprehensive review of nutritional strategies aimed at reducing injury risk and accelerating recovery, emphasizing the need for careful and evidence-based application. Effective approaches include increasing daily protein intake in conjunction with exercise, distributing protein evenly across meals, and prioritizing protein sources rich in leucine to support performance and recovery.

Creatine supplementation has also been shown to enhance strength and help preserve lean body mass during periods of immobilization and rehabilitation. Although the body of evidence is less extensive compared to creatine, growing interest has emerged around other nutrients such as fish oil, calcium, and vitamins C, D, and E for their potential role in attenuating muscle loss and inflammation during recovery. In particular, vitamin C supports collagen synthesis, tendon and ligament repair, and may improve post-surgical outcomes.

Additionally, *n*-3 fatty acids and curcumin have demonstrated promising effects in experimental models, including reductions in oxidative protein damage, neuronal injury, and inflammation, as well as the normalization of brain-derived neurotrophic factor (BDNF) and neurotransmitter levels. These findings suggest that such compounds may represent valuable nutritional support during the healing process (contribution 8).

Protein represents another key substrate during exercise. High-protein diets (HPDs) have gained increasing popularity due to their association with enhanced satiety and elevated energy expenditure, making them a common strategy in weight-loss interventions under caloric restriction [35]. In individuals with obesity, HPDs have been shown to promote reductions in body weight and improvements in metabolic health, including decreases in fasting insulin levels, waist circumference, and total fat mass.

In the context of resistance training (RT), HPDs are frequently adopted to support lean mass accretion and stimulate muscle protein synthesis [36]. However, high protein intake alone does not appear sufficient to significantly enhance muscle strength, although it is often associated with improvements in body composition [36]. In some cases, excessive protein intake may even have adverse effects, potentially contributing to increased inflammatory responses.

Supporting this, using a healthy rat model, Medeiros and colleagues demonstrated that an HPD led to impaired glucose tolerance, adipocyte hypertrophy, and increased inflammation in visceral adipose tissue after 12 weeks. Notably, resistance training mitigated these negative effects by restoring normal blood glucose levels and reducing adipose tissue inflammation, even in the context of a high-calorie diet. These findings suggest that resistance training exerts a protective effect against the potential metabolic and inflammatory consequences of high-calorie, high-protein diets. Additionally, resistance training was shown to increase reduced glutathione activity in adipose tissue regardless of dietary intake, highlighting its role in maintaining redox balance during exercise (contribution 9).

Muscle protein metabolism plays a fundamental role in maintaining proper tissue function. Muscle mass is regulated by the dynamic balance between muscle protein synthesis (MPS) and muscle protein breakdown (MPB). Resistance training (RT), when combined with adequate dietary protein intake, is widely recognized as an effective strategy to stimulate MPS, thereby promoting muscle hypertrophy and strength gains [37,38].

A growing body of research has also explored the combined effects of protein supplementation and bioactive compounds. In this context, Chen and colleagues reported that six weeks of supplementation with whey protein and vitamin D_3_, alongside resistance training in healthy young men, resulted in improvements in both muscle mass and strength. Notably, similar benefits were observed whether supplementation was consumed in the morning or before sleep, with no adverse effects on body composition. In contrast, participants undergoing resistance training with a non-nutritive placebo showed only moderate increases in one-repetition maximum (1RM) strength, without significant gains in muscle mass (contribution 10).

These adaptations appear to be largely driven by increases in MPS. While leucine-rich whey protein directly stimulates MPS, vitamin D may also contribute through both direct and indirect mechanisms. Specifically, vitamin D can enhance the expression of the vitamin D receptor (VDR) in muscle tissue and modulate circulating anabolic hormones. Serum levels of testosterone and insulin-like growth factor 1 (IGF-1), which are both closely linked to muscle mass, may play a role in amplifying the adaptive response to resistance training [39,40]. Moreover, both resistance training and vitamin D supplementation (individually or in combination) have been shown to increase testosterone and IGF-1 levels, particularly in individuals with vitamin D deficiency (contribution 10).

Endurance running is an accessible and cost-effective form of exercise that can be practiced over a wide range of distances, from recreational runs to ultramarathons, at both professional and amateur levels. It involves a substantial training load, even during the preparatory phase leading up to races [41]. However, research shows that simply increasing training volume does not guarantee improved performance and can instead lead to negative consequences, including oxidative, mechanical, and inflammatory stress, which may compromise both performance and overall health, such as by increasing injury risk [41]. Nutrition therefore represents a critical factor in training and race adaptation, playing a central role in the success of endurance running.

In recent decades, vegan (completely free of animal-derived foods) and vegetarian (meat-free) diets have grown in popularity for reasons, including health, athletic performance, ethics, and environmental concerns [42]. While the bioavailability of certain nutrients may be lower in vegan or vegetarian diets compared with omnivorous diets, plant-based diets are generally rich in carbohydrates and antioxidants, which can confer positive health effects [43].

In a study by Wirnitzer and colleagues, the performance and training characteristics of omnivorous, vegetarian, and vegan runners were compared. The results showed that omnivorous runners, compared to vegan and vegetarian runners, tended to train over longer preparation periods, relied more on supervised training, and completed a greater number of half-marathons and marathons with faster finishing times. Interestingly, there were no differences in weekly training volume among the three dietary groups. The authors suggest that these differences may reflect variations in motivations for participating in running events, as well as differences in health or fitness status (contribution 11).

In another study, Baroni and colleagues developed VegPlate for Sports, a vegetarian food guide (VFG) based on the validated VegPlate facilitation method and aligned with the Italian Dietary Reference Intakes (DRIs). VegPlate for Sports is designed for men and women who follow vegetarian diets (including lacto-ovo and vegan) and participate in sports, providing dietary planning tailored to individual body weight. This tool allows nutrition professionals to create meal plans that meet specific energy, carbohydrate, and protein requirements. Additionally, once clients or athletes are properly instructed, the platform can enable the self-management of plant-based diets that are nutritionally adequate, enjoyable, and varied (contribution 12).

A balanced and comprehensive diet is crucial for athletes, as it provides not only the appropriate amounts of macronutrients but also essential micronutrients that support cellular metabolism. Among these, selenium (Se) is an essential trace element (TME) for humans, which must be consumed in proper amounts because excessive intake can be toxic [44], while insufficient intake may exacerbate exercise-induced oxidative stress [45]. Although TME concentrations are generally tightly regulated through homeostatic mechanisms, selenium metabolism can be altered by exercise [45]. Most previous studies on Se have focused on supplementation with Se-rich products [46]. It has been suggested that athletes may require higher selenium intake to enhance the functionality and activity of endogenous antioxidant systems [45], and the adaptive response of these systems to training also depends on adequate nutritional support [46].

Among the key selenoproteins relevant in sports, the enzymes glutathione peroxidase (GPx) and glutathione reductase (GR) are particularly notable. These enzymes form the glutathione (GSH) redox cycle, a critical antioxidant system in the body [47].

An insightful study by Toro-Román and colleagues assessed both extracellular (serum, plasma, and urine) and intracellular (erythrocytes and platelets) selenium concentrations in athletes compared to sedentary individuals. They found that athletes exhibited lower intracellular Se levels, while extracellular concentrations showed no significant differences. This pattern may reflect both short- and long-term selenium depletion in the training group. Selenium is distributed to all tissues, including red blood cells, where it is stored after absorption [45]. When dietary selenium is insufficient for selenoprotein synthesis, these stores are mobilized, gradually depleting intracellular reserves.

These findings suggest that athletes engaged in regular physical training may require increased selenium intake to maintain optimal intracellular levels [45]. During periods of physiological stress, such as inflammation or exercise-induced oxidative stress, selenium is mobilized from intracellular compartments to support antioxidant defense, further depleting reserves and highlighting the need for adequate dietary selenium (contribution 13).

The timing of food intake plays a critical role during both training and competition phases. The interaction between exercise, nutrition, and metabolism is complex, making it a central focus for sports scientists and athletes alike. In this context, breakfast has often been highlighted for its potential effects on athletic performance [48,49]. In an insightful review by Stratton and colleagues, the authors note that the impact of breakfast consumption (or omission) varies greatly depending on the individual and context, and there is no universal approach. Breakfast can enhance, hinder, or have no effect on performance outcomes, depending on the type of sport, athlete characteristics, and specific goals. For instance, breakfast may influence acute performance more than it affects long-term adaptations to exercise or changes in body composition.

Overall, breakfast consumption consistently improved performance in morning endurance exercise lasting over 60 min, regardless of intensity, as well as in afternoon or evening tempo aerobic sessions (contribution 14). However, its effect on acute endurance training adaptations and resistance training outcomes appears minimal. Similarly, comparing breakfast consumption with omission did not show a significant impact on long-term gains in muscular performance. Some observed benefits were only seen in placebo-controlled trials, suggesting that psychological factors, rather than physiological ones, may contribute to performance changes.

The authors conclude that whether or not to consume breakfast should be guided by individual nutritional needs, as well as the goals of the training session and the overall program. Importantly, if an athlete chooses to skip breakfast, it is essential to ensure that daily macronutrient and micronutrient requirements are still met to prevent potential long-term negative effects on health (contribution 14).

Postprandial hyperglycemia and the resulting glycemic fluctuations have been shown to be stronger predictors of cardiometabolic disorders than chronic fasting hyperglycemia in individuals with type 2 diabetes [50]. Beyond pharmacological treatments, several lifestyle interventions can help reduce postprandial glycemic excursions, with physical exercise being one of the most effective strategies. Exercise has been demonstrated to improve glycemic control and the postprandial glycemic response not only in patients with diabetes, but also in healthy individuals [51].

In a follow-up study, Bellini and colleagues investigated the effects of different exercise modalities on the glycemic response to a standardized breakfast in individuals with type 2 diabetes. They found that aerobic exercise, resistance exercise, or a combination of both performed 30 min after the meal significantly improved postprandial glycemia. Aerobic exercise, whether performed alone or prior to resistance training, was most effective at reducing the postprandial glucose peak. Notably, even 15 min of circuit-based resistance training was sufficient to attenuate the glycemic response.

These findings have practical implications for prescribing and maintaining adherence to postprandial exercise programs in this population. Considering the cost-effectiveness and accessibility of different exercise types, brisk walking remains a simple yet highly effective option, particularly for older or untrained individuals. At the same time, resistance training (even in a circuit format) and combined exercise modalities are also viable strategies for postprandial metabolic control. This flexibility allows patients and exercise professionals to select exercise types based on individual abilities, preferences, and potential disease-related limitations (contribution 15).

One of the most intriguing nutritional strategies is the ketogenic diet. The low-carbohydrate ketogenic diet (LCKD) is characterized by high fat intake, moderate protein, and very low carbohydrate consumption—insufficient to meet normal metabolic demands [52]. An 8-week LCKD intervention has been shown to favorably modulate the expression of lipolytic and ketolytic genes, potentially enhancing performance during strenuous exercise [53]. Resistance training is also known to upregulate the fatty acid oxidation system, raising the question of whether combining a ketogenic diet with training could further improve exercise capacity.

In a study featured in this Special Issue, Ma and colleagues investigated this interaction by pairing a 10-week LCKD with an 8-week treadmill training program in an in vivo mouse model. The results indicated that while the ketogenic diet alone did not significantly enhance maximal exercise capacity, it did increase the expression of genes associated with fatty acid oxidation in oxidative muscle. These findings suggest that an LCKD may support endurance performance during moderate-intensity exercise, but it may not be the most effective strategy for high-intensity exercise (contribution 16).

Specific nutritional needs are influenced not only by the demands of a particular sport but also by the environment, including the climatic conditions in which physical activity takes place. For instance, the physiological adaptations of high-altitude populations to acute and chronic hypobaric hypoxia have been extensively studied, particularly regarding their metabolic and genetic mechanisms [54]. However, the role of nutrition in these contexts has received relatively little attention, despite its potential importance in supporting adaptation to altitude.

Over the past decades, numerous studies have highlighted the effects of high-altitude exposure on nutritional status, metabolism, and body composition [55]. Evidence indicates that altitude presents multiple physiological challenges, including weight loss (especially lean mass) and reduced fluid intake, driven by changes in dietary habits as well as metabolic effects induced by hypobaric hypoxia. Bondi and colleagues found that high-altitude excursions led to transient reductions in body fluids in both Western travelers and Himalayan porters, although the adaptation timelines differed between groups. These differences were influenced by variations in physical characteristics, acclimatization capacity, and diet. Additionally, the study reported decreases in muscle mass in both groups, even when protein intake was adequate, albeit relatively low. The authors concluded that a dietary approach that combines elements of both groups’ eating patterns could help minimize adverse changes in body composition for individuals traveling to high altitudes for sports or recreational purposes (contribution 17).

Assessment of body composition is widely used in sports to monitor athletes’ nutritional status and overall health [56,57]. Bioelectrical impedance analysis (BIA) and anthropometry are commonly employed as practical alternatives to reference techniques for evaluating body composition. In team sports, body fat percentage (FM%) is among the most informative parameters, and numerous predictive equations have been developed to estimate FM% using either BIA or anthropometric measurements. While it remains unclear which technique provides greater accuracy, the selection of an appropriate predictive equation appears to be a critical factor.

Campa and colleagues conducted a cross-sectional study involving 67 futsal players from Portugal’s top league, LIGA PLACARD (mean age 23.7 ± 5.4 years). They found that sport-specific predictive equations yielded valid FM% estimates, regardless of whether BIA or anthropometry was used. Estimates based on athletic equations closely matched those derived from DXA, whereas generalized equations were less accurate: BIA-based generalized equations tended to overestimate FM%, and anthropometry-based generalized equations underestimated it. The study concluded that both BIA and anthropometry can be used interchangeably for FM% assessment, provided that sport-specific predictive equations are applied (contribution 18).

Recent research has also highlighted a potential link between physical activity (PA) and the composition of the gut microbiota [58,59]. PA (including recreational activities, structured exercise, and sports) is recognized as a cornerstone of a healthy lifestyle and a key tool for preventing and managing various chronic diseases. Understanding how PA influences the gut microbiota could therefore reveal additional mechanisms by which exercise, independently or in combination with diet, impacts human health. Evidence indicates that certain microbial phyla and genera associated with exercise (particularly short-chain fatty acid (SCFA) producers) may help maintain intestinal epithelial homeostasis, increase mucus layer thickness, enhance metabolic and immune function, and modulate the gut–brain axis, potentially reducing neuroinflammation and mental fatigue. Conversely, exercise-induced stress may also lead to shifts in gastrointestinal microbiota composition [60,61].

In a notable systematic review, Dorelli and colleagues reported that physical activity can increase the abundance of beneficial gut bacteria while limiting the growth of potentially harmful species. In particular, most studies comparing athletes and sedentary individuals observed greater diversity and higher levels of Firmicutes, such as Lactobacilli, in athletes. These findings were less consistent in studies involving the general population, likely reflecting differences in exercise volume between athletes and non-athletes. This suggests that the quantity of physical activity may be an important factor in shaping gut microbiota composition (contribution 19).

Taken together, the data presented in the articles published in the Special Issue “The Role of Nutrition in Exercise and Sport” in the journal *Nutrients* offer significant new perspectives on the role of personalized nutrition based on the type of exercise performed, diet/supplementation and the environmental conditions under which physical activity occurs. All of these have specific nutritional requirements to support physical activity, and thus inevitably result in a modulation of the microbiota, which can significantly impact the athlete’s well-being and fitness. New and more comprehensive/innovative research are warranted to better shed light on the role of nutrition in these contexts.

## Figures and Tables

**Figure 1:**
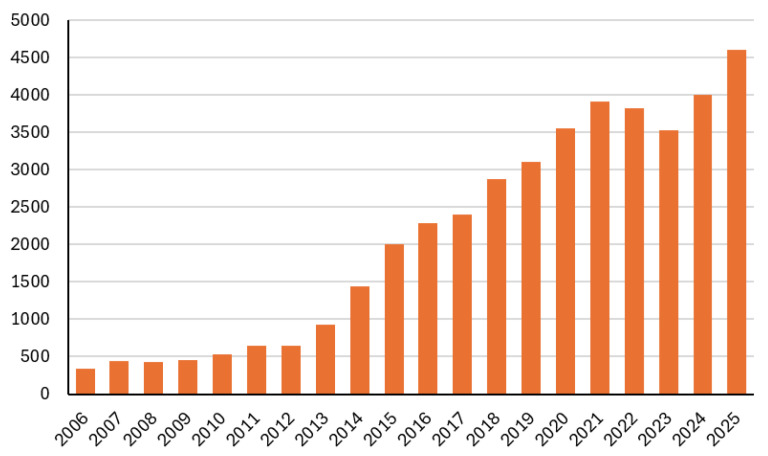
Number of publications indexed in PubMed from 2006 to 2025 applying the keyword “sport” in combination with “nutrition”.
